# Spectroscopic Studies on Nicotine and Nornicotine in the UV Region†

**DOI:** 10.1002/chir.22141

**Published:** 2013-03-14

**Authors:** Peter M Clayton, Carl A Vas, Tam TT Bui, Alex F Drake, Kevin McAdam

**Affiliations:** 1 British American Tobacco, Group R&DRegents Park Road, Southampton, SO15 8TL, UK; 2 Applied Photophysics Limited21 Mole Business Park, Leatherhead, KT22 7BA, UK

**Keywords:** nicotine, nornicotine, spectroscopy, UV, ECD, electronic circular dichroism

## Abstract

The UV absorption and electronic circular dichroism (ECD) spectra of *(R)*- and *(S)*-nicotine and (*S*)-nornicotine in aqueous solution were measured to a significantly lower wavelength range than previously reported, allowing the identification of four previously unobserved electronic transitions. The ECD spectra of the two enantiomers of nicotine were equal in magnitude and opposite in sign, while the UV absorption spectra were coincidental. In line with previous observations, *(S)*-nicotine exhibited a negative cotton effect centered on 263 nm with vibronic structure (π–π_1_* transition) and a broad, positive ECD signal at around 240 nm associated with the *n*–π_1_* transition. As expected this band disappeared when the pyridyl aromatic moiety was protonated. Four further electronic transitions are reported between 215 and 180 nm; it is proposed the negative maxima around 206 nm is either an *n*–σ* transition or a charge transfer band resulting from the movement of charge from the pyrrolidyl N lone pair to the pyridyl π* orbital. The pyridyl π–π_2_* transition may be contained within the negative ECD signal envelope at around 200 nm. Another negative maximum at 188 nm is thought to be the pyridyl π–π_3_* transition, while the lowest wavelength end-absorption and positive ECD may be associated with the π–π_4_* transition. The UV absorption spectra of *(S)*-nornicotine was similar to that of *(S)*-nicotine in the range 280–220 nm and acidification of the aqueous solution enhanced the absorption. The ECD signals of (*S*)-nornicotine were considerably less intense compared to *(S)*-nicotine and declined further on acidification; in the far UV region the ECD spectra diverge considerably. *Chirality 25:288–293, 2013*. © 2013 Wiley Periodicals, Inc.

## INTRODUCTION

**Fig. 1:**
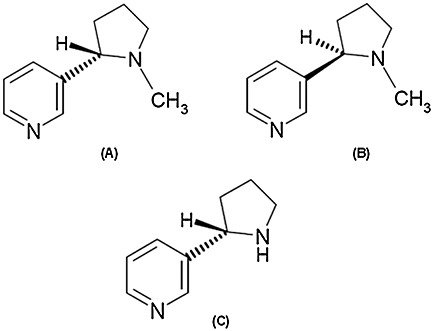
Molecular structure of *(S)*-(−)-nicotine (**A**) and *(R)*-(+)-nicotine (**B**) and *(S)*-(−)-nornicotine (**C**).

The pyridine alkaloid nicotine, [1-methyl-2-(3-pyridyl) pyrrolidine], and its *N*-demethylated analogue, nornicotine, exists as two mirror image isomers or enantiomers (Fig. 1). *(S)*-(−)-nicotine [CAS 54-11-5] is the dominant isomer present in tobacco^1^ (genus *Nicotiana*) and tobacco smoke.^2^ *(R)*-(+)-nicotine [CAS 25162-00-9] is present in only small amounts (0.2 − 1%) in raw and processed tobacco,^1^ while in tobacco smoke the prevalence of *(R)*-(+)-nicotine is higher and has been measured at 2–3% of the total amount of nicotine present.^2^,^3^ The temperature-induced racemization of nicotine in a tobacco matrix has recently been explored,^4^ while pure nicotine under conditions of open-tube pyrolysis exhibits considerable stereochemical stability with respect to temperature.^5^ Nornicotine (Fig. 1(C) is a secondary tobacco alkaloid produced by the enzymatic *N*-demethylation of nicotine.^6^ The amount of nornicotine [CAS 5746-86-1] present in tobacco is typically less than 5% of the total alkaloid content,^6^ but in contrast to nicotine, nornicotine extracted from tobacco is racemic. 7

Nicotine has been reported to have numerous effects on the central nervous system^8^ (CNS) and is notably an agonist at nicotinic acetylcholine receptors; *(S)*-(−)-nicotine is six times more potent at muscle-type nicotinic acetylcholine receptors compared to *(R)*-(+)-nicotine.^9^ The stereochemical configuration of the carbon atom at the 2’ position of the pyrrolidine ring therefore has an important impact on the biological activity of this alkaloid.^10^

Nornicotine is also an agonist at nicotinic acetylcholine receptors albeit it has been reported racemic nornicotine has lower affinity for [^3^H]nicotine-sensitive neuronal receptors than *(S)*-nicotine.^11^ The pharmacological properties of the enantiomers of nornicotine are less well characterized than those of nicotine in part due to the non-availability of a single isomer from plant sources. A recent examination into the antinociceptive properties of nornicotine enantiomers reported that the analgesic properties resided predominantly in the *(S)* isomer; in contrast various defined side effects were more pronounced with the *(R)*-enantiomer.^12^ An examination of the spectroscopic and chiroptical properties of the pyridine alkaloids is a valuable addition to the current state of knowledge.

In this article, the electronic circular dichroism (ECD) and UV absorption spectra of the enantiomers of nicotine and *(S)*-nornicotine are reported between 300 and approximately 190 nm at the pH adopted in aqueous solution and following the addition of acid. A number of the electronic transitions in the far UV region of both alkaloids were tentatively assigned to the spectra. We believe this is the first time that the ECD and UV absorbance spectra of the enantiomers of nicotine and nornicotine at wavelengths below 220 nm have been described and preliminarily assigned.

## EXPERIMENTAL PROCEDURE

### Chemicals

*(S)*-(−)-nicotine, ERM® - AC802b, was purchased from LGC Standards (Teddington, UK), declared total purity (m/m): 99.65% (gravimetrically determined).

*(R)*-(+)-nicotine was purchased from Toronto Research Chemical (Toronto, Canada), declared chemical purity: 97% (NMR), quoted specific rotation: (+117° dm^−1^ cm^3^ g^−1^, 589 nm).

*(S)*-nornicotine was synthesized at Target Molecules (Southampton, UK) following the method described by Joyce and Leete^13^ involving the oxidation of *(S)*-nicotine to its *N*-1’-oxide followed by *N*-demethylation with ferric nitrate. The chemical purity was determined as 97% (GC).

The enantiomeric purity of *(S)*-nornicotine was greater than 99%, as determined by chiral GC analysis using a dimethylated cyclodextrin GC column,^14^ the kind gift of Prof. V. Schurig, University of Tübingen. The details of the chromatographic method will be published elsewhere.

Perchloric acid (99%) used in ECD measurements was purchased from Sigma-Aldrich (Gillingham, UK) and diluted to 20% in double-distilled water.

Perchloric acid (99%) used in UV absorption study (double beam instrument) was purchased from Sigma-Aldrich (Gillingham, UK) and diluted to 20% in deionized water.

(*R,S*)-nornicotine used in UV absorption (double beam instrument) was purchased from Toronto Research Chemical, declared chemical purity: 97% (TLC).

### Equipment

#### Chiroptical spectroscopy

Simultaneous ECD and UV spectra were measured using a Chirascan-plus spectropolarimeter (Applied Photophysics Ltd, Leatherhead, UK).

The pH of solutions were measured using a Corning pH105 pH meter equipped with a ThermoRussel K series electrode (Russell Mainstream Supply Ltd, Fife, UK).

#### UV absorption spectroscopy

UV spectra were also obtained using a double beam Cary-Varian 5E spectrophotometer.

The pH of solutions were measured using a Mettler-Toledo EasySeven pH meter equipped with an InLab413 electrode (Leicester, UK).

### Methodology

#### Nicotine chiroptical spectroscopy

Solutions of *(S)*-(−)-nicotine (30.6 µg/ml) and *(R)*-(+)-nicotine (30.8 µg/ml) were prepared in deionized water (18.2MΩ.cm resistivity). The pH of the *(S)*-(−)-nicotine solution was pH 8.68 and for *(R)*-(+)-nicotine solution was pH 8.88 (20 °C).

The UV absorbance and the ECD spectra of the nicotine enantiomers were measured in the wavelength region of 300 nm to 180 nm in a 0.5-mm pathlength rectangular strain-free cell. Strain-free fused silica cells were utilized to ensure artefact-free ECD spectra. The optical beam of the single beam instrument was continuously flushed with pure evaporated nitrogen gas, which allowed access to the low wavelengths. The spectra were recorded with a 0.5-nm wavelength step size, 1-sec measurement time-per-point, and the spectral bandwidth was 1 nm. The spectra were water baseline corrected and measured at 20 °C.

ECD spectra of nicotine enantiomers were also obtained in the wavelength region of 300 nm to 190 nm, using a 10-mm pathlength rectangular strain-free cell. The spectra were recorded with a 0.5-nm wavelength step size, 1-sec measurement time-per-point, and the spectral bandwidth was 1 nm. The spectra were water baseline corrected, and measured at 20 °C. (The UV cutoff wavelength of a 10-mm aqueous solution at 298 K has been determined at 185 nm^15^; in a 0.5-mm pathlength cell the cutoff will be at a lower wavelength.)

An aliquot of 30.6 µg/ml *(S)*-(−)-nicotine solution was basified by the micro-addition of aqueous sodium hydroxide to give a solution of pH 10.15. Further micro-aliquots of aqueous hydrochloric acid produced solutions of pH 5.67 and 2.08 (final concentration 30 µg/ml). The ECD and UV absorption spectra were measured in the wavelength region of 300 nm to 200 nm in a 10-mm pathlength rectangular strain-free cell. The spectra were recorded with a 0.5-nm wavelength step size, 1-sec measurement time-per-point, and the spectral bandwidth was 1 nm. The spectra were water baseline corrected, and measured at 20 °C.

The ECD and UV absorption spectra of a separate aqueous solution of *(S)*-nicotine (13.8 µg/ml, pH 8.20, 20 °C) was measured between 300 nm and 190 nm in a 5-mm pathlength cell. This solution was acidified by the micro-addition of 20% perchloric acid to produce a solution was of pH 1.74. The use of perchloric acid confers the advantage that it is transparent in far UV region (below 210 nm) of the spectrum^16^ compared to hydrochloric acid.

#### Nicotine UV absorption spectroscopy

A solution of *(S)*-(−)-nicotine (30 µg/ml) was prepared in deionized water (18.2 MΩ.cm), the pH of the solution was 8.25 (22 °C). The solution was acidified by the micro-addition of 20% perchloric acid to produce a solution of pH 2.38, 22 °C). UV absorption spectra were measured between 300 nm and 200 nm using a 10-mm pathlength cell and a double beam spectrophotometer.

#### Nornicotine chiroptical spectroscopy

A solution of *(S)*-(−)-nornicotine (14.0 µg/ml) was prepared in deionized water (18.2 MΩ.cm). The pH of the solution was pH 9.10 (20 °C). This solution was acidified (pH 1.98, 20 °C) by the micro-addition of 20% perchloric acid. ECD spectra were measured between 300 nm and 190 nm in a 5-mm pathlength cell, using a single beam spectropolarimeter.

#### Nornicotine UV absorption spectroscopy

A solution of *(S)*-(−)-nornicotine (30 µg/ml) was prepared in deionized water (18.2 MΩ^.^cm), the pH of the solution was 8.81 (22 °C). The solution was acidified by the micro-addition of 20% perchloric acid to produce a solution of pH 2.44 (22 °C). UV absorption spectra were measured between 300 nm and 200 nm, using a 10-mm pathlength cell and a double beam spectrophotometer.

## RESULTS AND DISCUSSION

### ECD and UV Absorption Spectra of Nicotine Enantiomers

**Fig. 2:**
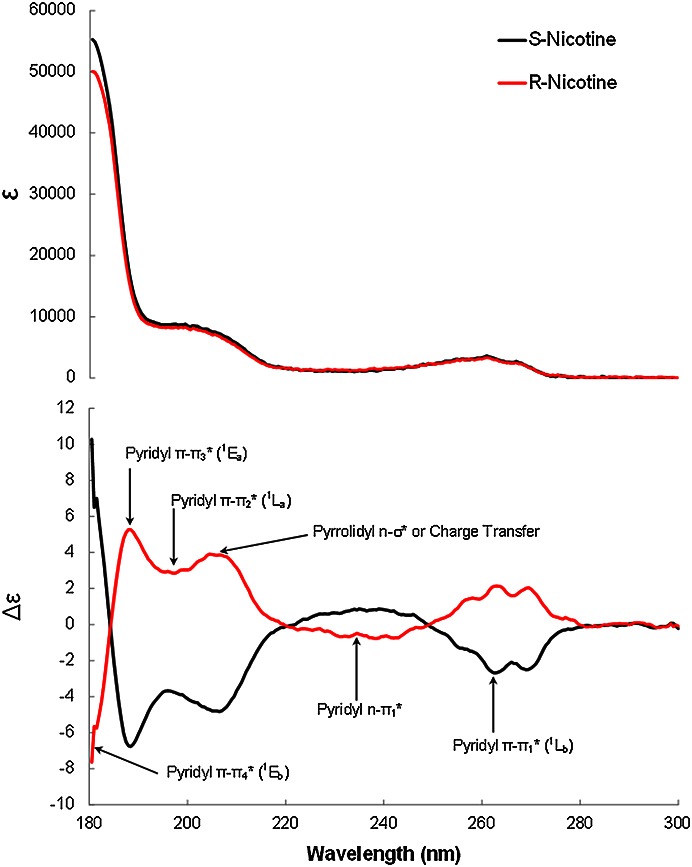
Spectropolarimetric UV absorption and ECD spectra of *(S)*-(−)-nicotine (30.6 µg/ml, pH 8.68) and *(R)*-(+)-nicotine (30.8 l µg/ml, pH 8.88) in water showing suggested spectral assignments at 20 °C. The pathlength was 0.5 mm.

The ECD spectra of *(S)*-(−)-nicotine and *(R)*-(+)-nicotine together with UV absorbance spectra (at pH 8.68 and pH 8.88, respectively) are shown in Figure 2. The ECD and UV absorbance spectra were measured simultaneously using the single-beam Chirascan-plus spectrometer. As expected, the ECD spectra of the two enantiomers of nicotine were almost exact mirror images of one another, while the UV absorbance spectra were almost indistinguishable from each other. The minor differences in UV absorbance observed at the low-wavelength end probably arise as a result of small differences in the impurity profile of the enantiomers. The general features and spectroscopic assignment of UV spectra of organic chromophores are described elsewhere.^17^

The ECD spectra show a negative cotton effect at around 263 nm for *(S)*-(−)-nicotine and the positive cotton effect for *(R)*-(+)-nicotine. The negative ECD band at 263 nm for *(S)*-(−)-nicotine has been observed previously^18^–^20^ and has been attributed to the π–π_1_* or ^1^L_b_ transition of the pyridine moiety.^18^,^20^ A notable feature of this transition, observable in both the ECD and UV absorption spectra, is the existence of vibronic fine structure which is a result of simultaneous changes involving both electronic and vibrational energies of the aromatic moiety.

The positive ECD signal at around 240 nm for *(S)*-(−)-nicotine is associated with the *n*–π_1_* transition. This transition is a result of the movement of charge from the pyridyl N lone pair (nonbonding electrons) to an antibonding molecular orbital (π_1_*). This feature of the ECD spectrum has been attributed to the *n*–π_1_* transition of the pyridine moiety.^18^,^20^ The *n*–π_1_* transition is electric dipole forbidden (very weak absorption, ε < 100) but is magnetic dipole allowed thereby ensuring a significant ECD.

The ECD maximum at around 206 nm is the result of an n-σ* transition, but there is also the possibility of charge transfer from the pyrrolidyl N lone pair to the pyridyl π*. Both these transitions would be expected to vanish upon protonation of the pyrrolidyl group (p*K*_a_ ∼8).^18^

The π–π_2_* (or ^1^L_a_) transition of the pyridine moiety underlies the ECD signal at around 200 nm. The ECD maxima at 188 nm may be the pyridyl π–π_3_* (^1^E_a_) transition.^21^ The lowest wavelength absorption and ECD is just about visible at 180 nm (peak maxima is not observed); this may be associated with the π–π_4_* (or ^1^E_b_) transition.^21^

**Fig. 3:**
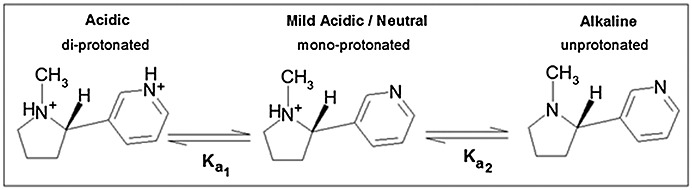
Acid–base equilibria of *(S)*-nicotine in aqueous solution.

**Fig. 4:**
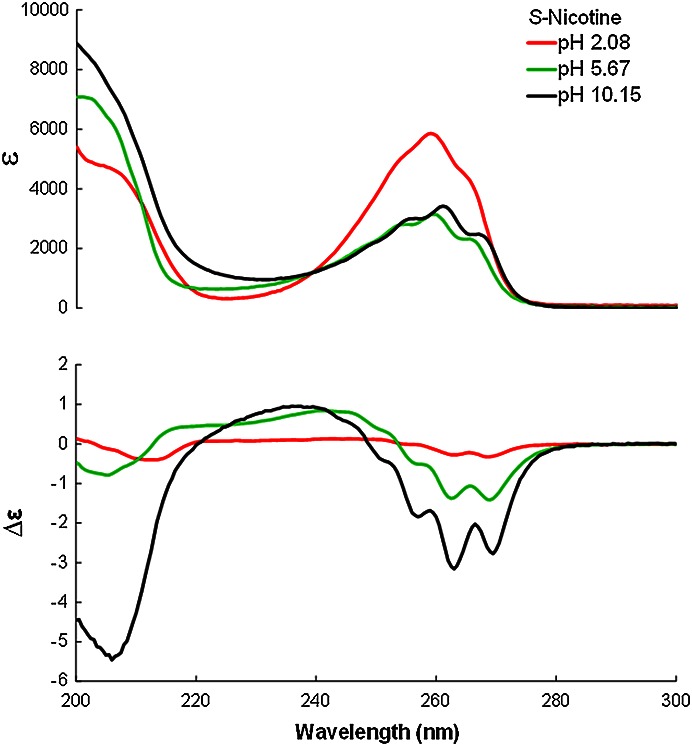
Spectropolarimetric UV absorption and ECD spectra of *(S)*-(−)-nicotine (30 µg/ml) at pH 2.08, 5.67, 10.15; the pathlength was 10 mm.

In aqueous solution, both nicotine and nornicotine contain two nitrogen atoms capable of accepting a proton and will undergo the equilibria shown in Figure 3 (shown for nicotine). In aqueous solution the heterocyclic pyrrolidine nitrogen is considerably more basic than the pyridine nitrogen, and the acid ionization constants (p*K*_a_) of nicotine have been determined as 2.96 (p*K*_a1_, 20 °C) and 8.07 (p*K*_a2_, 20 °C).^22^ ECD and UV absorption spectra of *(S)*-(−)-nicotine (30 µg/ml) were measured at pH 2.08, pH 5.67, and pH 10.15, and are shown in Figure 4. At these pH values, nicotine will be predominantly present in di-protonated, mono-protonated, and unprotonated forms, respectively.

From Figure 4 it is apparent that the positive ECD band associated with *n*–π_1_* transition centered between 235 and 243 nm vanishes at acidic pH when the pyridyl moiety is protonated; as expected, this transition exhibits minimal absorption.^17^ The absorption associated with the π–π_1_* transition increases substantially on acidification, similar to the UV absorption spectrum of pyridine.^21^ The ECD signal associated with the pyrrolidyl lone pair, at around 206 nm, decreases when the pyrrolidyl moiety is protonated at pH values below ∼ pH 8.^22^

### Comparison of Absorption and ECD Spectra for S-(−)-Nicotine with Previous Literature

Absorption and ECD spectra for nicotine are shown graphically in Figures 4, and Figure 6. Table 1 summarizes the measurements of the UV absorption spectrum of *S*-(−)-nicotine between 300 and 220 nm from Testa and Jenner^18^ and Swain et al.^23^ compared to this publication; the results show good concurrency.

**TABLE 1:** Comparison of UV absorption spectra of aqueous *S*-(−)-nicotine

Solvent pH	λ maximum (π–π_1_*)	Molecular extinction coefficient ε_λ_	Source of data
pH 1	X	5500	Ref. 18
pH 2.08	259 nm	5864	Fig. 4
pH 2.38	259 nm	5328	Fig. 6
45 mM aq. HCl	259 nm	5530	Ref. 23
pH 5.36	X	3000	Ref. 18
pH 5.67	260 nm	3140	Fig. 4
Water	260 nm	3020	Ref. 23
pH 8.25	260 nm	2994	Fig. 6
pH 10.15	261 nm	3422	Fig. 4

X: unable to determine/not reported. The unit of **ε** is l cm^–1^ mol^–1^.

The divergence in ECD data, shown in Table 2, is almost certainly related to the less favorable signal-to-noise ratio of the instrumentation available at the time, which relied on electro-optical crystal (Pockel cell) technology. The ECD data presented by Testa and Jenner show broad concurrence with the spectra presented here; certainly the signs of the Cotton effects were identical. The ECD spectral characteristics presented by Atkinson et al.^19^ are in reasonable agreement with spectra presented here; however, changes in the pH of solutions examined will be sources of difference in themselves.

**TABLE 2:** Comparison of ECD spectral data of aqueous *S*-(−)-nicotine

Solvent pH	λ maximum (π–π_1_*)	[θ]_λ_	λ_0_	λ maximum (*n*–π_1_*)	[θ]_λ_	Source of data
pH 5.36 buffer	263	−3600	255 nm	240 nm	+4000	Ref. 18
pH 5.67	263	−4544	254 nm	242 nm	+2776	Fig. 4
pH 7.2 buffer	264	−6330	247 nm	236 nm	+3420	Ref. 19
pH 8.2 buffer	264	−6440	249 nm	240 nm	+2500	Ref. 19
pH 8.68	263	−8848	249 nm	239 nm	+2864	Fig. 2
pH 10.15	263	−10,421	249 nm	236 nm	+3157	Fig. 4
pH 11.5 buffer	263	−5300	251 nm	240 nm	+3600	Ref. 18

The unit of [θ] is deg^.^cm^2^ dmol^–1^, λ_0_ is the wavelength at which the CD signal is equal to zero (crosses the abscissa).

### ECD and UV Absorption Spectra of (S)-Nornicotine

**Fig. 5:**
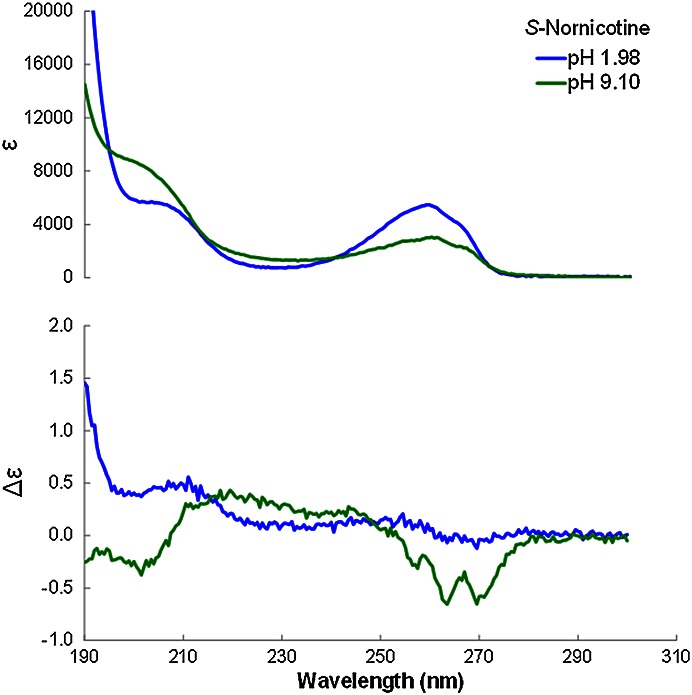
Spectropolarimetric UV absorption and ECD spectra of *(S)*-(−)-nornicotine (14.0 µg/ml) at pH 9.10 and 1.98; 5 mm pathlength.

The ECD and absorption spectra of *(S)*-(−)-nornicotine at pH 9.10 and pH 1.98 are shown in Figure 5. We believe this is the first depiction of the full ECD spectrum to 190 nm of an enantiomer of nornicotine in the scientific literature.

Table 3 shows the ECD data for *S*-(−)-nornicotine reported here compared to literature source.^18^ Similar to the ECD of (*S*)-nicotine, the acidification of (*S*)-nornicotine solution results in a diminution of the ECD signal.

**TABLE 3:** Comparison of ECD spectral data of aqueous *S*-(−)-nornicotine

Solvent pH	λ maximum (π–π_1_*)	[θ]_λ_	λ_0_	λ maximum (*n*–π_1_*)	[θ]_λ_	Source of data
pH 6.10 buffer	263 nm	−3300	252 nm	242 nm	+2300	Ref. 18
pH 1.98	∼270 nm	∼ − 400	263 nm	∼244 nm	∼ + 600	Fig. 5
pH 11.5 buffer	261 nm	−4300	250 nm	241 nm	+2300	Ref. 18
pH 9.10	263 nm	−2163	253 nm	∼244 nm	∼ + 900	Fig. 5

The unit of [θ] is deg^.^cm^2^ dmol^–1^, λ_0_ is the wavelength at which the CD signal is equal to zero (crosses the abscissa).

### UV Absorption Spectra of Nicotine and Nornicotine

**Fig. 6:**
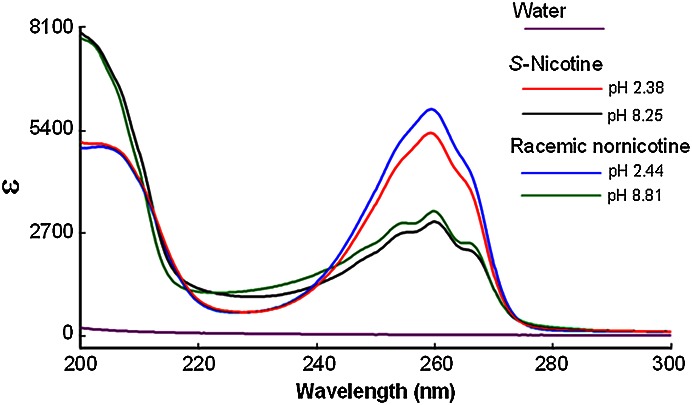
Spectrophotometric UV absorption spectra of *(S)*-nicotine (30 µg/ml) at pH 8.25 and pH 2.38 and racemic nornicotine (30 µg/ml) at pH 8.81 and pH 2.44; the pathlength was 10 mm.

It is possible to obtain UV spectra from an ECD spectropolarimeter (Chirascan), which is a single-beam instrument. Conceptually, double-beam instruments are better suited for the measurement of UV absorption spectra.^24^ For this reason, UV absorption spectra of nicotine, nornicotine, and acidified solutions were measured using a Cary-Varian 5E spectrophotometer, shown in Figure 6.

Table 4 summarizes the measurements of the UV absorption spectrum of *S*-(−)-nornicotine between 300 and 220 nm in this publication; a small bathochromic shift in the π –π_1_* absorption band is observed in nornicotine with increasing pH similar to nicotine shown in Table 1. The absorption of nornicotine associated with the π–π_1_* transition centered at 260 nm increases substantially on acidification, similar to the UV absorption spectrum of pyridine.^21^ This phenomenon is a property attributable to the protonation of the pyridyl moiety, and this feature is also observed in nicotine (Fig. 4).

**TABLE 4:** Comparison of UV sbsorption spectra of aqueous nornicotine

Solvent pH	λ maximum (π–π_1_*)	Molecular extinction coefficient ε_λ_	Source of data
pH 1.98	259 nm	5475	Fig. 5
pH 2.44	259 nm	5439	Fig. 6
pH 9.10	260 nm	3054	Fig. 5
pH 8.81	260 nm	2989	Fig. 6

The unit of **ε** is l cm^–1^ mol^–1^.

## CONCLUSION

In this article aqueous nicotine ECD and UV absorption spectra are presented between 300 and 180 nm, a significantly lower wavelength than reported previously in the literature. Additionally the ECD and UV absorption spectra of *(S)*-(−)-nornicotine are also presented between 300 and 200 nm.

The spectra of *(S)*-nicotine were previously described to a minimum wavelength of approximately 210 nm and two transitions at 263 and 240 nm were assigned: the negative signal centered on 263 nm showing vibronic fine structure was attributed to the π–π_1_* transition, while the positive band at 240 nm was assigned to the pyridyl *n*–π_1_* transition.^18^,^19^ The assignment of *n*–π_1_* transition is supported by the disappearance of this band upon protonation of the pyridyl moiety at pH 2.

In this work, four previously unobserved electronic transitions of nicotine between 215 and 180 nm are described. The negative maximum around 206 nm is either an *n*–σ* transition or a charge transfer band resulting from the movement of charge from the pyrrolidyl N lone pair to the pyridyl π* orbital. There is good evidence that this transition involves the pyrrolidyl lone electron pair because the band disappears in acidic solution when the pyrrolidyl moiety is protonated. The band associated with the pyridyl π–π_2_* transition may be confined within the negative ECD signal envelope at around 200 nm. Another negative maximum at 188 nm is thought to be pyridyl π–π_3_* transition, while the lowest wavelength end-absorption and positive ECD (∼180 nm) may be associated with the π–π_4_* transition. Transitions that are sensitive to pH may be of use for determining the ionisation status of nicotine in aqueous solution.^22^

There is limited published data on the spectroscopic properties of nornicotine. As expected, the UV absorption spectrum of nornicotine is analogous to that of nicotine at ∼ pH 9 and in acidic solution. However, the ECD spectra of the *S*-isomers of both compounds show considerable divergence in intensity, but not in wavelength maxima. At ∼260 nm both compounds exhibit a negative ECD with vibronic fine structure. This band is considerably less intense in the case of *(S)*-nornicotine. The *n*–π_1_* transition at ∼240 nm of both compounds diminishes greatly following solution acidification. It is noteworthy the band at 206 nm, thought to arise from the *n*–σ* transition or a charge transfer band, is negative for *(S)*-nicotine but is ill defined for *(S)*-nornicotine.

These observations encapsulate that nicotine and nornicotine enantiomers are similar, but not identical, in their spectroscopic properties.

## References

[1] Armstrong DW, Wang X, Ercal N (1998). Enantiomeric composition of nicotine in smokeless tobacco, medicinal products and commercial products. Chirality.

[2] Perfetti TA, Coleman WM (1998). Chiral gas chromatography selected ion monitoring mass selective detection analysis of tobacco material and tobacco smoke. Beitr Tabakforsch Int.

[3] Perfetti TA, Coleman WM, Smith WS (1998). Determination of mainstream and sidestream cigarette smoke components for cigarettes of different tobacco types and a set of reference cigarettes. Beitr Tabakforsch Int.

[4] Liu B, Yao W, Su Q (2008). Racemization of S-(−)-nicotine during smoking and its relationship with pyrolysis process. J Anal Pyrol.

[5] Clayton P, Lu A, Bishop L (2010). The pyrolysis of (−)-*(S)*-nicotine: racemization and decomposition. Chirality.

[6] Siminszky B, Gavilano L, Bowen SW, Ralph E, Dewey RE (2005). Conversion of nicotine to nornicotine in *Nicotiana tabacum* is mediated by CYP82E4, a cytochrome P450 monooxygenase. Proc Nat Acad Sci.

[7] Crooks PA, Gorrod JW, Jacob P (1999). Chemical properties of nicotine and other tobacco-related compounds. Analytical determination of nicotine and related compounds and their metabolites.

[8] Silvette H, Hoff EC, Larson PS, Haag HB (1962). The actions of nicotine on central nervous system function. Pharmacol Rev.

[9] Rozental R, Aracava Y, Scoble GT, Swanson KL, Wonnacott S, Albuquerque EX (1989). Agonist recognition site of the peripheral acetylcholine receptor ion channel complex differentiates the enantiomers of nicotine. J Pharmacol Exp Ther.

[10] Barlow RB, Hamilton JT (1965). The stereospecificity of nicotine. Br J Pharmacol.

[11] Damaj MI, Fei-Yin M, Dukat M, Glassco W, Glennon RA, Martin BR (1998). Antinociceptive responses to nicotinic acetylcholine receptor ligands after systemic and intrathecal administration in mice. J Pharmacol Exp Ther.

[12] Holtman JR, Crooks PA, Johnson-Hardy JK, Wala EP (2010). The analgesic and toxic effects of nornicotine enantiomers alone and in interaction with morphine in rodent models of acute and persistent pain. Pharmacol Biochem Behav.

[13] Joyce NJ, Leete E (1989). The formation of 1-methyl-3-nicotinoylpyrrolidine from nicotine-1’-oxide. Heterocycles.

[14] Schurig V (2010). Use of derivatized cyclodextrins as chiral selectors for the separation of enantiomers by gas chromatography. Ann Pharmaceut Francaise.

[15] Fox MF (1973). Far ultraviolet solution spectrophotometry. Appl Spectrosc.

[16] Fritz JS, Gjerde DT (2009). Ion chromatography.

[17] Mason SF (1961). Molecular electronic absorption spectra. Quart Rev Chem Soc.

[18] Testa B, Jenner P (1973). Circular dichroic determination of the preferred conformation of nicotine and related chiral alkaloids in aqueous solution. Mol Pharmacol.

[19] Atkinson WM, Han SM, Purdie N (1984). Determination of nicotine in tobacco by circular dichroism spectropolarimetry. Anal Chem.

[20] Tomati A, Ochiai E, Hirai H, Makishima S (1966). The effect of hydrogen chloride on the optical rotation of nicotine. J Org Chem.

[21] Rosini C, Bertucci C, Pini D, Salvadori P, Soccolini F, Delogu G (1983). The vacuum ultraviolet-circular dichroism spectrum of an isolated pyridine chromophore. J Chem Soc Chem Commun.

[22] Clayton PM, Vas CA, Bui TTT, Drake AF, McAdam K (2013). Spectroscopic investigations into the acid–base properties of nicotine at different temperatures. Anal Methods.

[23] Swain ML, Eisner A, Woodward CF, Brice BA (1949). Ultraviolet absorption spectra of nicotine, nornicotine and some of their derivatives. J Am Chem Soc.

[24] Drake AF (1986). Polarisation modulation—the measurement of linear and circular dichroism. J Phys E Sci Instrum.

